# Prevalence and risk factors for cervical neoplasia: a cervical cancer screening program in Beijing

**DOI:** 10.1186/1471-2458-14-1185

**Published:** 2014-11-19

**Authors:** Lixin Tao, Lili Han, Xia Li, Qi Gao, Lei Pan, Lijuan Wu, Yanxia Luo, Wei Wang, Zihe Zheng, Xiuhua Guo

**Affiliations:** School of Public Health, Capital Medical University, Beijing, China; Beijing Municipal Key Laboratory of Clinical Epidemiology, Beijing, China; Beijing Obstetrics and Gynecology Hospital, Beijing, China; School of Medical Science, Edith Cowan University, Perth, Australia

**Keywords:** Cervical cancer, Screening program, Risk factor, High-grade cervical squamous intraepithelial lesions

## Abstract

**Background:**

Cervical cancer is the second most common cancer and cause of cancer-related death for women worldwide. The aims of this study were to investigate the prevalence of cervical neoplasia and examine factors associated with high-grade cervical squamous intraepithelial lesions (HSIL) among women taking part in a cervical cancer screening program in Beijing.

**Methods:**

Women aged 25–65 years were screened using the ThinPrep cytologic test and gynecologic examination. Univariate and multivariate logistic regressions were conducted to investigate factors associated with HSIL.

**Results:**

Among 728,704 women screened, the prevalence of cervical intraepithelial neoplasia (CIN) I, II, III was 50.2, 34.0, and 36.4 per 100,000, respectively. Prevalence of cervical cancer was 12.2 per 100,000. Risk factors for HSIL included being in age group of 46–55 years (adjusted odds ratio [aOR] = 1.15, 95% CI: 1.07–1.44, compared with the 25–35 age group), bleeding after intercourse (aOR = 2.08, 95% CI: 1.40–3.10), and presence of trichomonas vaginalis infection (aOR = 2.62, 95% CI: 1.35–5.07), cervical inflammation (aOR = 4.22, 95% CI: 3.39–5.26), and genital warts (aOR = 3.89, 95% CI: 2.54–7.70). High education level (college and above compared with junior middle school or lower) was found to be protective (aOR = 0.79, 95% CI: 0.37–0.90).

**Conclusions:**

The prevalence of cervical neoplasia is relatively high in Beijing. Women aged 46–55 years, those with a lower education level, those reporting bleeding after intercourse, and those affected by *Trichomonas vaginalis* infection, cervical inflammation and genital warts are at higher risk for HSIL. Particular efforts should be made to ensure these women are included in cervical cancer screening programs.

## Background

Cervical cancer is the second most common cancer and cause of cancer-related death in women worldwide[1, 2]. Cervical intraepithelial neoplasia (CIN) refers to changes in squamous cells of the cervix, where more extensive changes (CIN grades II and III) are known as high-grade squamous intraepithelial lesions (HSIL). At least 25% of women with HSIL will progress to carcinoma in situ or invasive cancer if lesions are left untreated[3]. The identification of risk factors for HSIL is of pivotal importance.

Evidence suggests that human papillomavirus (HPV) infection and health-related lifestyles influence the progression of CIN. HPV plays a crucial role in the development of cervical cancer and the precursor lesions. HPV is essential to the transformation of cervical epithelial cells, particularly subtypes 16 and 18[4–6]. However, only a minority of women infected with HPV progress to CIN or HSIL. Therefore, cofactors that aid viral persistence and disease progression must exist.

A positive association between smoking and cervical cancer has consistently been observed, across different geographic regions[7–9]. Dose–response associations with smoking intensity and duration were detected in a previous study[10]. However, adenocarcinoma of the cervix, which usually accounts for less than 10% of all cervical cancers, has no significant association with smoking[11]. Increased risk of invasive cervical cancer with exposure to passive smoking during adulthood has been demonstrated[12, 13]. Indoor exposure to cooking oil fumes is associated with CIN[3], while cooking in kitchens equipped with fume extractors and keeping extractors on while cooking can protect women from this risk[14].

In recent years, strong evidence from many epidemiologic and experimental studies has emerged, demonstrating that inflammation also plays an important role in the development of CIN and cervical cancer[15, 16]. An association between the use of anti-inflammatory drugs and reduced risk of cancer, as well as a decrease in precancerous lesions, was shown in epidemiologic studies[16, 17].

Factors relating to sexual behavior have also been linked to cervical cancer and its precursors. Studies have demonstrated that bacterial vaginosis and *Trichomonas vaginalis* infection are significantly associated with persistent HPV infection and the development of cervical cancer[18–20]. Many studies have also suggested that women with multiple sexual partners are at high risk for HPV acquisition and cervical cancer[21, 22]. Oral contraceptive use is associated with the development of cervical cancer[23–25]. A systematic review of hormonal contraceptive use reported that the risk of in situ cervical cancer increased even for women with less than 5 years’ hormonal contraceptive use, but the risk of invasive cervical cancer increased only after 5 years’ use. The risk for both conditions declined with time since last use of hormonal contraceptives, and there was no elevated risk for invasive cervical cancer 10 years since the end of exposure[26].

The cervical cancer screening program was free for 25 to 65-year-old women permanently residing in Beijing in 2009. To the best of our knowledge, there has been no similar, large-scale cervical cancer screening program conducted in Beijing prior to this. The aims of the present study were to estimate prevalence of cervical neoplasia and explore potential risk factors for HSIL among women living in Beijing.

## Methods

### Subjects

This study was conducted in 2009 as part of a national program offering free cervical and breast cancer screening for 25 to 65-year-old women permanently residing in Beijing. All women aged 25–65 years who were willing to participate in the screening program were included, with the exception of those with a history of uterine sarcoma, fallopian tube tumors, uterine fibroids, benign ovarian tumors, ovarian cancer, vulvar carcinoma, vulvar malignant melanoma, organ transplant or cancer treatment. Women were informed of the purpose of the screening program and gave their signed, informed consent prior to enrolment. The study was approved by the Ethics Committee of Beijing Obstetrics and Gynecology Hospital, Capital Medical University.

### Data collection

Data were collected by cervical cancer screening case record cards. The cards recorded socio-demographic information, reproductive history and sexual behavior, medical history, gynecologic examination history, and TCT and histologic test results.

A technical manual was designed to standardize the screening process, and medical staff were trained prior to commencement of the program. All gynecologic examinations were supervised and gynecologists conducted each examination blinded to the results of the TCT test. If TCT results were abnormal, subjects were referred for histologic testing to determine CIN grade and to receive relevant treatment. Approximately 10% of gynecologic examinations were double-checked by experts and the coincident diagnosis rate was 95%. Additionally, screening experts checked 20% of the positive results and 10% of the negative results. Approximately 5% of all record cards were randomly checked and the proportion with errors or omissions was less than 5%.

Socio-demographic information recorded for each woman included age, education level, occupation, and address. Information was also collected on reproductive history and sexual characteristics, including length of menstrual cycle, duration of menstrual period, gravidity, parity, number of abortions, contraceptives used, reported bleeding after intercourse, and detection of *Trichomonas vaginalis* infection, yeast infection, bacterial vaginosis, cervical inflammation, and genital warts. Medical history included any history of uterine sarcoma, fallopian tube tumors, uterine fibroids, ovary benign tumors, ovarian cancer, vulvar carcinoma, vulvar malignant melanoma, organ transplants, or cancer treatment.

### Outcome variables

The primary study outcome was CIN grade, categorized as: normal, CIN I, CIN II, CIN III, and cervical cancer. HSIL was defined as CIN grades II and III.

### Statistical analysis

Distributions of participants’ characteristics were examined by rank-sum test, *χ*^2^, or Fisher’s exact test, as appropriate. Participants with CIN II and CIN III grades were combined as a HSIL group for subsequent univariate and multivariate logistic regression analysis to assess the association between HSIL and each potential risk factor. Data were analyzed using SAS (version 9.2; SAS Institute, Chicago, IL, USA) and ArcGIS (ArcGIS 10; ESRI Inc., Redlands, CA, USA).

## Results

**Figure 1:**
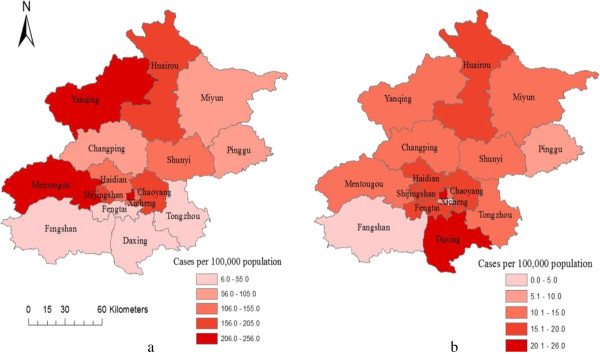
**The prevalence of a) HSIL and b) cervical cancer in 18 districts of Beijing.** Abbreviation: HSIL = high-grade squamous intraepithelial lesion.

**Figure 2:**
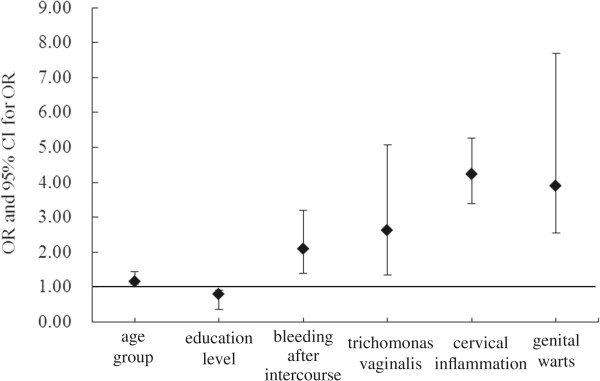
**Adjusted OR and 95% CI for risk factors for HSIL.** Abbreviation: HSIL = high-grade squamous intraepithelial lesion; age group (46–55 vs 25–35); education level (college and above vs junior middle school or lower).

A total of 728,704 women from the 18 districts of Beijing participated in the screening program, representing 9.4% of the 25 to 65-year-old female population in Beijing in 2009. Of these participants, 366 women (50.2 per 100,000) were diagnosed as CIN I, 248 (34.0 per 100,000) as CIN II, 265 (36.4 per 100,000) as CIN III and 89 (12.2 per 100,000) as having cervical cancer. Prevalence of HSIL (CIN grades II and III) was 70.40 per 100,000 women. The prevalence of HSIL and cervical cancer by Beijing district are shown in Table 1 and Figure 1. Yanqing district had the highest prevalence of CIN I, CIN II, and HSIL, at 136.8, 119.0, and 255.8 per 100,000, respectively. The highest prevalence of CIN III was in Mentougou district (149.3 per 100,000) and the highest prevalence of cervical cancer was in Xicheng district (25.3 per 100,000).There were significant differences between women with HSIL (n = 513) and those with normal histology (n = 702,168) in terms of age group, education level, parity, bleeding after intercourse, contraceptive used, presence of *Trichomonas vaginalis* infection, cervical inflammation, and genital warts. However, there was no significant difference in terms of work or not, menstrual cycle, menstrual period, gravidity, number of abortions, and presence of yeast infection and bacterial vaginosis (Table 2). Risk factors significantly associated with presence of HSIL in univariate logistic regression were being in age band of 46–55 years (compared with the reference group 25–35 years), higher education level, parity, bleeding after intercourse, and presence of *Trichomonas vaginalis*, cervical inflammation and genital warts (Table 3). Risk factors that remained significant in multivariate logistic regression were being in the age band of 46–55 years (adjusted odds ratio [aOR] = 1.15, 95% CI: 1.07–1.44, compared with the age band of 25–35 years), bleeding after intercourse (aOR = 2.08, 95% CI: 1.40–3.10), and presence of *Trichomonas vaginalis* infection (aOR = 2.62, 95% CI: 1.35–5.07), cervical inflammation (aOR = 4.22, 95% CI: 3.39–5.26) and genital warts (aOR = 3.89, 95% CI: 2.54–7.70). Higher education level was found to be protective against HSIL (aOR = 0.79, 95% CI: 0.37–0.90, college and above compared with junior middle school or lower education level) (Table 4, Figure 2).

**Table 1:** **The prevalence of CIN and cervical cancer**

Districts	Total	CIN I [n (/100,000)]	CIN II [n (/100,000)]	CIN III [n (/100,000)]	HSIL(CIN II/III) [n (/100,000)]	Cervical cancer [n (/100,000)]
Dongcheng	13194	6 (45.5)	3 (22.7)	4 (30.3)	7 (53.1)	1 (7.6)
Xicheng	27649	23 (83.2)	25 (90.4)	30 (108.5)	55 (198.9)	7 (25.3)
Chongwen	8382	3 (35.8)	3 (35.8)	5 (59.7)	8 (95.4)	0 (0.0)
Xuanwu	18787	1 (5.3)	3 (16.0)	11 (58.6)	14 (74.5)	1 (5.3)
Chaoyang	121141	128 (105.7)	71 (58.6)	69 (57.0)	140 (115.6)	25 (20.6)
Haidian	83442	27 (32.4)	27 (32.4)	21 (25.2)	48 (57.5)	4 (4.8)
Fengtai	63816	8 (12.5)	7 (11.0)	5 (7.8)	12 (18.8)	9 (14.1)
Shijingshan	25987	19 (73.1)	9 (34.6)	19 (73.1)	28 (107.8)	5 (19.2)
Mentougou	13395	8 (59.7)	3 (22.4)	20 (149.3)	23 (171.7)	1 (7.5)
Fangshan	72004	22 (30.6)	8 (11.1)	4 (5.6)	12 (16.7)	1 (1.4)
Daxing	53898	5 (9.3)	5 (9.3)	5 (9.3)	10 (18.6)	13 (24.1)
Tongzhou	26183	0 (0.0)	0 (0.0)	2 (7.6)	2 (7.6)	2 (7.6)
Shunyi	72194	32 (44.3)	22 (30.5)	19 (26.3)	41 (56.8)	6 (8.3)
Pinggu	30937	8 (25.9)	6 (19.4)	7 (22.6)	13 (42.0)	1 (3.2)
Huairou	46360	43 (92.8)	32 (69.0)	15 (32.4)	47 (101.4)	8 (17.3)
Miyun	22953	7 (30.5)	2 (8.7)	5 (21.8)	7 (30.5)	2 (8.7)
Changping	11570	3 (25.9)	2 (17.3)	1 (8.6)	3 (25.9)	1 (8.6)
Yanqing	16812	23 (136.8)	20 (119.0)	23 (136.8)	43 (255.8)	2 (11.9)
Total	728704	366 (50.2)	248 (34.0)	265 (36.4)	513 (70.4)	89 (12.2)

*Abbreviations*: *CIN* cervical intraepithelial neoplasia, *HSIL* high-grade squamous intraepithelial lesions.

**Table 2:** **Basic characteristics of subjects**

Variables	Normal	HSIL	/100,000		***P***
Age group				26.93	<0.0001
25-35	76694	56	73.0		
36-45	201001	151	75.1		
46-55	283055	247	87.2		
56-65	141418	59	41.7		
Education level				7.89	0.0193
Junior middle school or lower	468032	365	77.9		
High school or technical secondary school	156502	110	70.2		
College and above	77634	38	48.9		
Work or not				0.77	0.3807
Yes	487062	365	74.9		
No	215106	148	68.8		
Bleeding after intercourse				12.47	0.0004
No	682097	485	71.1		
Yes	20071	28	139.3		
Contraceptive used				195.00	<0.0001
Condom	340067	186	54.7		
Contraceptive pills	11551	34	293.5		
Intrauterine device use	196821	196	99.5		
Security period	981	3	304.9		
Coitus interruptus	7955	25	313.3		
No contraceptive measures	144793	69	47.6		
Trichomonas vaginalis				−	0.0140
No	697064	504	72.3		
Yes	5104	9	176.0		
Yeast infection				−	0.7595
No	698372	510	73.0		
Yes	3796	3	79.0		
Bacterial vaginosis				−	0.5268
No	698835	510	72.9		
Yes	3333	3	90.0		
Cervical inflammation				191.77	<0.0001
No	658979	406	61.6		
Yes	43189	107	247.1		
Genital warts				−	0.0013
No	699972	506	72.2		
Yes	2196	7	317.8		
Menstrual cycle	29 (28–30)^*a*^	29 (28–30)^*a*^	.	0.67^*b*^	0.4140 ^*b*^
Menstrual period	5 (4–6)^*a*^	5 (4–7)^*a*^	.	2.97^*b*^	0.0851^*b*^
Gravity	2 (1–3)^*a*^	2 (2–3)^*a*^	.	0.16^*b*^	0.6902^*b*^
Parity	1 (1–2)^*a*^	1 (1–1)^*a*^	.	18.55^*b*^	<0.0001^*b*^
Number of abortion	1 (1–2)^*a*^	1 (0–2)^*a*^	.	2.77^*b*^	0.0963^*b*^
Total	702168	513			

*Abbreviations: HSIL* high-grade squamous intraepithelial lesions. Trichomonas vaginalis is defined as vaginalis infected by trichomonas. Yeast infection is defined as vaginosis infected by yeast. Bacterial vaginosis is defined as vaginosis infected by bacterial. Cervical inflammation is defined as moderate or serious inflammation.

^*a*^: Median (P25-P75).

^*b*^: Rank sum test.

−: Fisher’s exact test.

**Table 3:** **Risk factors for HSIL assessed by univariate logistic regression**

Variables	OR	95% CI for OR		***P***
		Lower	Upper	
Age group				
25-35	Ref	Ref	Ref	Ref
36-45	1.37	0.82	1.84	0.6521
46-55	1.15	1.01	3.98	0.0302
56-65	0.57	0.40	1.12	0.3601
Education level				
Junior middle school or lower	Ref	Ref	Ref	Ref
High school or technical secondary school	1.68	0.98	2.04	0.3112
College and above	0.54	0.10	0.87	0.0261
Work or not				
Yes	Ref	Ref	Ref	Ref
no	1.03	0.85	1.25	0.7356
Contraceptive used				
Condom	Ref	Ref	Ref	Ref
Contraceptive pills	1.15	0.64	2.06	0.9484
Intrauterine device use	1.29	0.94	1.59	0.9442
Hypoderm contraceptive implants	1.13	0.88	2.14	0.9559
Security period	0.59	0.19	1.85	0.9720
Coitus interruptus	1.42	0.83	1.97	0.9406
No contraceptive measures	0.90	0.68	1.20	0.9568
Bleeding after intercourse				
No	Ref	Ref	Ref	Ref
Yes	2.60	1.75	3.86	<0.0001
Trichomonas vaginalis				
No	Ref	Ref	Ref	Ref
Yes	2.55	1.32	4.93	0.0055
Yeast infection				
No	Ref	Ref	Ref	Ref
Yes	1.14	0.37	3.54	0.8248
Bacterial vaginosis				
No	Ref	Ref	Ref	Ref
Yes	1.31	0.42	4.08	0.6389
Cervical inflammation				
No	Ref	Ref	Ref	Ref
Yes	4.27	3.45	5.28	<0.0001
Genital warts				
No	Ref	Ref	Ref	Ref
Yes	4.41	2.82	6.19	<0.0001
Menstrual cycle	1.00	0.99	1.01	0.9911
Menstrual period	1.02	0.97	1.08	0.4526
Gravity	0.99	0.92	1.06	0.7287
Parity	0.74	0.65	0.86	<0.0001
Number of abortions	0.98	0.63	1.53	0.9225

*Abbreviations*: *HSIL* high-grade squamous intraepithelial lesions, *OR* odds ratio, *CI* confidence interval.

**Table 4:** **Risk factors for HSIL assessed by multivariate logistic regression**

Variables	OR	95% CI for OR		***P***
		Lower	Upper	
Age group				
25-35	Ref	Ref	Ref	Ref
36-45	1.46	0.88	1.98	0.2143
46-55	1.15	1.07	1.44	0.0254
56-65	0.78	0.52	1.16	0.4399
Education level				
Junior middle school or lower	Ref	Ref	Ref	Ref
High school or technical secondary school	1.59	0.90	1.96	0.2416
College and above	0.79	0.37	0.90	0.0337
Bleeding after intercourse				
No	Ref	Ref	Ref	Ref
Yes	2.08	1.40	3.10	0.0003
Trichomonas vaginalis				
No	Ref	Ref	Ref	Ref
Yes	2.62	1.35	5.07	0.0043
Cervical inflammation				
No	Ref	Ref	Ref	Ref
Yes	4.22	3.39	5.26	<0.0001
Genital warts				
No	Ref	Ref	Ref	Ref
Yes	3.89	2.54	7.70	<0.0001

*Abbreviations*: *HSIL* high-grade squamous intraepithelial lesions, *OR* odds ratio, *CI* confidence interval.

## Discussion

This study represents the first large-scale cervical cancer screening program in Beijing. Women in 18 districts aged 25–65 years and with a registered, permanent Beijing address were eligible to participate. However, only 9.4% of eligible women were evaluated. The prevalence of CIN I, II, III, and cervical cancer was 50.2, 34.0, 36.4, and 12.2 per 100,000, respectively. Identified risk factors for HSIL in this population were being in the age group of 46–55 years, bleeding after intercourse, presence of *Trichomonas vaginalis* infection, cervical inflammation, and genital warts, while higher education was found to be protective.

Prevalence of precancerous cervical lesions (CIN grades I–III) in this study population (0.12%) was lower than that reported in 2009 (0.20%)[27]. Prevalence of cervical cancer was 12.2 per 100,000, which is comparable to rates reported elsewhere: 16.2 per 100,000 and 10.3 per 100,000 reported from more developed countries, and 19.1 per 100,000 reported from less developed countries[28]. While the study population in this study is predominantly urban, there appears to be little difference in rates of cervical cancer between urban and rural regions in China. According to China Cancer Registration Annual Report 2004, in which 43 cancer registries were included, the incidence of cervical cancer was 5.3 per 100,000 in urban and 4.9 per 100,000 in rural areas[29].

Supporting results from previous studies, being aged 46–55 years and having a lower educational attainment level were identified as significant risk factors for HSIL in our study. A community-based screening program in Hong Kong similarly found that women aged 40–49 years and women having received primary school education only were at increased risk of cervical abnormalities[30]. Other studies have also identified older age, high gravidity, and low educational status as significant risk factors for development of cervical cancer[31–34].

Our results show that women who reported bleeding after intercourse had a higher risk of developing cervical epithelial abnormality. This association has been identified previously[35] and is not surprising, as bleeding after intercourse may indicate vaginal infections, cervical dysplasia, or uterine fibroids, all of which may lead to cervical abnormalities.

There was a significant association between an abnormal TCT test and having a history of gynecologic infections. *Trichomonas vaginalis* infection was associated with a high relative risk of HSIL. This supports previous findings: a meta-analysis of 24 studies examining the association between *Trichomonas vaginalis* infection and cervical neoplasia (including both CIN and cervical cancer) found a significant positive association[36]. There is some epidemiologic evidence to suggest that genital tract disease such as cervical inflammation might be linked to cervical cancer or high-grade lesions[37, 38], and a significant association between cervical inflammation and HISL was identified in this study. *Trichomonas vaginalis* may act as a cofactor facilitating the development of cervical HPV infection to high-grade lesion and cervical cancer[39, 40]. Multiple studies have demonstrated an association between previous and current *Trichomonas vaginalis* infection and cervical dysplasia and human papillomavirus[41–43]. A history of genital warts has been reported as a good predictor of risk for carcinoma in situ[44] and the similar association was detected in the present study. The association between genital warts and HISL is likely due to concurrent infection with different HPV subtypes[45, 46]. It is also probably related to multiple sex partners[47].

There are some limitations to this study. First, the authors did not receive information on many other lifestyle factors that may potentially be associated with development of cervical abnormalities. Information on smoking, alcohol consumption and other lifestyle factors is required to explore their association with HSIL. Second, because of budget limitations, detection of HPV infection was not included in this study, which is crucial in the development of CIN and cervical cancer. Despite this, the current study, benefitting from a large sample size, provides valuable information for the assessment of CIN and cervical cancer.

## Conclusions

The prevalence of cervical neoplasia was relatively high. Women in the age group of 46–55 years, those with lower educational attainment, those reporting bleeding after intercourse, and those suffering from *Trichomonas vaginalis* infection, cervical inflammation and genital warts are at high risk for HSIL. Particular efforts should be made to ensure these women are included in cervical cancer screening programs.

## Abbreviations

HSIL: High-grade squamous intraepithelial lesions
CIN: Cervical intraepithelial neoplasia
aOR: Adjusted odds ratio
CI: Confidence interval
HPV: Human papillomavirus
TCT: ThinPrep cytologic test.
